# Neuronal intranuclear inclusion disease presenting with recurrent dizziness and headache: a case report with 5-year follow-up

**DOI:** 10.3389/fgene.2025.1719182

**Published:** 2025-12-08

**Authors:** Limin Li, Xinghua Luan, Kai Liu, Chengzhe Wang

**Affiliations:** 1 Department of Neurology, Songjiang Hospital Affiliated to Shanghai Jiao Tong University School of Medicine, Shanghai, China; 2 Department of Neurology, Shanghai Sixth People’s Hospital Affiliated to Shanghai Jiao Tong University School of Medicine, Shanghai, China; 3 Department of Radiology, Songjiang Hospital Affiliated to Shanghai Jiao Tong University School of Medicine, Shanghai, China

**Keywords:** neuronal intranuclear inclusion disease, dizziness, headache, NOTCH2NLC, diffusion weighted imaging

## Abstract

Neuronal intranuclear inclusion disease (NIID) is a highly heterogeneous chronic neurodegenerative disease characterized by ataxia, extrapyramidal symptoms, peripheral neuropathy, autonomic nervous symptoms, and cognitive dysfunction. So far, recurrent dizziness and headache have been reported in cases of NIID. We report a case of a 71-year-old female patient with NIID, who was followed up for 5 years. The primary manifestations in this patient were episodic dizziness and headache, which are relatively nonspecific symptoms. Due to these characteristics, it is easy to misdiagnose or overlook the disease at the initial diagnosis. The patient exhibited characteristic high signal intensity at the corticomedullary junction on diffusion-weighted imaging (DWI) sequences of the head magnetic resonance imaging (MRI). We diagnosed NIID after detecting round, non-enveloped filamentous structures with a diameter of 1–2 μm within the nuclei of fibroblasts and Schwann cells and revealing 114 repeats of GGC amplification at the 5′end of the NOTCH2NLC gene. The findings of this case study indicate that patients presenting with atypical symptoms should be considered for this disease.

## 1 Introduction

Neuronal intranuclear inclusion disease (NIID) is a chronic neurodegenerative disorder triggered by the irregular expansion of GGC repeats in the NOTCH2NLC gene. Its hallmark is the presence of eosinophilic intranuclear inclusions in the cells of the nervous system, internal organs, and skin tissues. Historically, NIID was classified into infantile, juvenile, and adult types, and further divided into familial and sporadic forms. While the classic clinical triad involves dementia, muscle weakness, and autonomic dysfunction, recent large-scale cohort studies have significantly expanded the known clinical spectrum. It is now recognized that NIID can present with “episodic neurogenic events” or “paroxysmal symptoms,” including recurrent headache, encephalitic episodes, and stroke-like attacks, which may occur in up to one-third of patients (Tai et al., 2023; Tian et al., 2022). However, because these nonspecific symptoms often mimic common conditions like migraine or cerebrovascular disease, NIID is prone to misdiagnosis, particularly when typical neurological deficits are absent. Currently, cranial magnetic resonance imaging (MRI) showing high signal intensity at the corticomedullary junction on diffusion-weighted imaging (DWI), combined with skin biopsy and genetic testing, facilitates accurate diagnosis (Sone et al., 2016; Sone et al., 2011). Here, we report a 5-year follow-up of a patient in whom recurrent dizziness and headache were the sole manifestations. We aim to highlight the diagnostic challenges posed by such atypical, paucisymptomatic presentations and to discuss the observed dissociation between radiological progression and clinical stability.

## 2 Case report

A 71-year-old female presented with a 7-year history of recurrent dizziness and headache. The patient has experienced episodic dizziness and headaches for a total of 7 years. She was followed regularly at our hospital for the most recent five-year period, during which three serial MRI scans were performed. The dizziness was characterized as non-vertiginous, manifesting as a persistent sensation of light-headedness and disequilibrium. The patient denied any illusion of self or environmental motion (vertigo), and the symptoms were not position-dependent. The headache was located in the right temporal, parietal, and occipital regions, presenting as paroxysmal, ultrashort, stabbing pains, each lasting for several seconds. The attacks occurred in irregular bouts, approximately 5–6 times per year. The headache was not associated with migrainous features (photophobia, phonophobia, nausea) or cranial autonomic symptoms (Author anonymous, 2018). Previous symptomatic treatments with loxoprofen and flunarizine provided limited and transient relief.

A systematic diagnostic workup was conducted to exclude other potential causes of chronic dizziness and headache in this elderly individual. Cerebrovascular assessment via Magnetic Resonance Angiography (MRA) of the head revealed no evidence of significant stenosis, occlusion, or malformation of the major intracranial arteries, making large-vessel vasculopathy an unlikely cause for the symptoms. Cardiac evaluation included a 12-lead electrocardiogram (ECG), which was normal, showing a regular sinus rhythm without evidence of arrhythmia that could lead to cerebral hypoperfusion. An echocardiogram was not performed, as there were no clinical signs or ECG findings suggestive of a cardioembolic source. Systemic evaluation through comprehensive laboratory investigations (including complete blood count, serum electrolytes, renal and liver function panels, and glucose) was unremarkable. Thyroid function tests revealed subclinical hypothyroidism (TSH 15.1 uIU/mL; normal free T4), which was considered an incidental finding, as the patient’s specific presentation of stabbing headache and the pathognomonic MRI findings were not consistent with a thyroid-related etiology.

**FIGURE 1:**
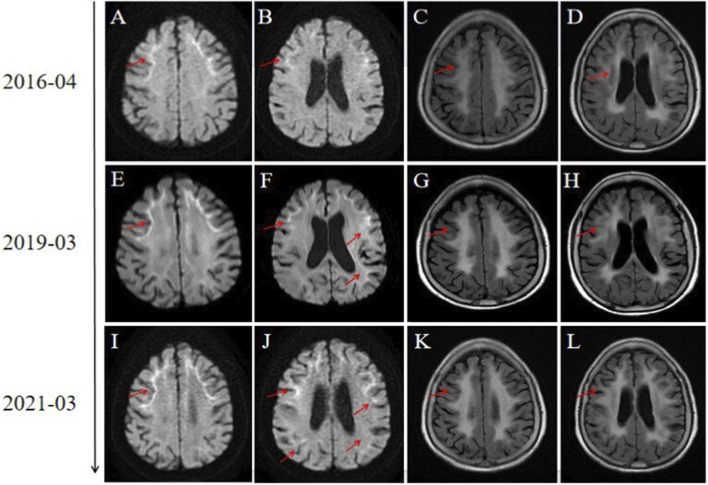
Head MRI findings in neuronal intranuclear inclusion disease (NIID). Panels **(A–D)**, **(E–H)**, and **(I–L)** correspond to the DWI and the corresponding T2/FLAIR images of the patient in April 2016, March 2019, and March 2021, respectively. Panels **(A,B)**, **(E,F)**, and **(I,J)** display symmetric, line-like high signals on diffusion-weighted imaging (DWI), indicative of the “insular ribbon sign” (red arrow). Panels **(C,D)**, **(G,H)**, and **(K,L)** show extensive white matter lesions on T2/FLAIR (red arrow). Comparative findings reveal disease progression from 2016 to 2021.

On neurological examination, motor, sensory, and cerebellar functions were unremarkable. Formal cognitive screening yielded a Mini-Mental State Examination (MMSE) score of 28/30 and a Montreal Cognitive Assessment (MoCA) score of 26/30. These scores indicate grossly preserved global cognitive function, although subtle executive dysfunction was suggested by points lost in the visuospatial/executive domain on the MoCA. Neuroelectrophysiological studies, including nerve conduction studies, were not performed during the evaluation. A cranial MRI conducted during the first visit revealed curvilinear high signals in the bilateral cortical-subcortical junctions, particularly in the frontal lobe, on diffusion-weighted imaging (DWI), and T2 FLAIR sequences showing diffuse symmetric high signals in the bilateral white matter (Figure 1). We performed three imaging assessments over 5 years, which showed significant progression of the imaging signs (Figure 1), but the patient’s symptoms had not significantly changed compared to the initial onset.

After this extensive evaluation failed to identify a common cause for her chronic symptoms, the highly characteristic finding of high signal intensity at the corticomedullary junction on DWI led to the consideration of NIID. Based on these imaging findings, after obtaining the patient’s consent, a skin biopsy and genetic testing were further conducted. The skin electron microscopy examination revealed fibrous structures with a diameter of 1–2 μm, round and unencapsulated, in the nuclei of fibroblasts and Schwann cells (Supplementary Figure S1). Guanine–cytosine-rich PCR (GC-PCR) and repeat-primed polymerase chain reaction (RP-PCR) gene testing showed 114 GGC repeat sequences in the NOTCH2NLC gene (Supplementary Figure S2). Genetic testing for the NOTCH2NLC GGC repeat expansion was performed on the patient’s two adult sons, and both results were negative for the pathogenic expansion. A detailed three-generation family history was obtained, which was negative for any individuals with relevant neurological symptoms, such as dementia, ataxia, or movement disorders. The patient’s parents are deceased and were thus unavailable for genetic testing.

The subsequent confirmation by skin biopsy and definitive genetic testing makes NIID the most plausible diagnosis to explain the patient’s full clinical and radiological picture. While a coincidental co-occurrence of primary headache and NIID cannot be absolutely excluded, the principle of parsimony suggests a single underlying diagnosis is more likely, especially given the emerging recognition of headache as a feature of NIID. Based on these findings, the patient was diagnosed with adult-onset, apparently sporadic NIID.

## 3 Discussion

NIID, first reported by Lindenberg et al. (1968), is a highly heterogeneous chronic neurodegenerative disease. While historically defined by dementia and motor symptoms, recent large-cohort studies have established that headache is a common feature and part of a distinct clinical subtype. For example, a study of 223 NIID patients by Tai et al. reported headache in 24.7% of the total cohort. Notably, this study proposed an “episodic neurogenic event-dominant type” as a major subtype accounting for 32.3% of patients, which explicitly includes episodic headaches (Tai et al., 2023). Similarly, a study by Tian et al. on 247 patients found that 66.8% had paroxysmal symptoms, with 10.9% experiencing chronic headaches (Tian et al., 2022). More recently, Zhong et al. noted headache as a symptom in 35.6% of their cohort of 45 patients (Zhong et al., 2025). These findings collectively demonstrate that headache is a core component of the NIID phenotype. A key distinguishing feature of the present case is the specific headache semiology and its clinical course. The headache in the reported patient is characterized as paroxysmal stabbing pain, whereas much of the literature describes migraine-like features. For instance, the case reported by Xie et al. described a patient who presented with “severe throbbing headache” preceded by distinct visual and sensory auras, a presentation highly consistent with migraine with aura (Xie et al., 2022). The heterogeneity of headache phenotypes within NIID-encompassing both classic migraine-like attacks and non-migrainous stabbing headaches-may reflect the variable anatomical distribution and functional impact of neuronal inclusions within central pain pathways.

The dissociation between progressive MRI abnormalities and stable clinical symptoms over a five-year period is a key finding of this report. This phenomenon is critical for understanding NIID pathophysiology, and two non-mutually exclusive mechanisms are proposed: First, the progressive MRI changes may represent an early, functionally compensated neuropathological stage. The high signal on DWI in NIID is pathologically correlated with spongiotic changes in the subcortical white matter, a process linked to severe astrocyte dysfunction and depletion in those specific areas, while T2-FLAIR hyperintensities correspond to diffuse myelin pallor and axonal loss (Yokoi et al., 2016). These pathological processes can advance over years, as seen in the serial imaging of this case. However, overt clinical deficits may not manifest until the pathological burden surpasses the brain’s capacity for functional compensation, a concept known as neural reserve. This principle is well-established in other chronic neurodegenerative diseases, such as Alzheimer’s disease, where a significant amyloid plaque burden can accumulate for years before the onset of clinical dementia (Sperling et al., 2011). The patient in this report may be in a prolonged paucisymptomatic stage where this reserve is sufficient to maintain neurological function despite the advancing white matter pathology. Second, a topographical dissociation likely exists between the prominent MRI lesions and the neural substrates of the patient’s specific symptoms. The striking MRI abnormalities are predominantly located in the supratentorial white matter. However, the patient’s stable symptoms of dizziness and stabbing headache may originate from dysfunction in regions where pathology is less apparent on conventional MRI, such as the brainstem, vestibular nuclei, and trigeminal sensory pathways. Landmark pathological studies in NIID have definitively confirmed the widespread presence of intranuclear inclusions in these very structures, including the pontine nuclei and cranial nerve nuclei (Sone et al., 2016). It is plausible that microscopic inclusion pathology in these critical brainstem networks is sufficient to generate her persistent but mild symptoms, while the extensive supratentorial white matter damage has not yet crossed the threshold to cause detectable cognitive or motor deficits.

The diagnosis of NIID in this patient was established in full accordance with contemporary diagnostic criteria, despite the atypical clinical presentation. A cornerstone of the diagnostic process was the presence of the pathognomonic radiological hallmark on neuroimaging - a linear high-intensity signal at the corticomedullary junction on diffusion-weighted imaging (DWI), a feature considered highly specific for NIID. While abnormal nerve conduction studies (NCS) are frequently reported, particularly in patients with the limb weakness-dominant subtype, they are not an indispensable criterion for diagnosis. Indeed, the absence of significant peripheral neuropathy in our case is consistent with the expanding clinical spectrum of NIID, where core diagnostic features can secure a diagnosis even when peripheral signs are not prominent (Sone et al., 2016). The diagnosis was further substantiated at a cellular level by skin biopsy, which revealed characteristic filamentous intranuclear inclusions on electron microscopy, a well-validated and crucial antemortem diagnostic finding (Sone et al., 2011). Ultimately, genetic analysis provided unequivocal confirmation by identifying a pathogenic expansion of 114 GGC repeats in the NOTCH2NLC gene, a repeat number well within the established pathogenic range (Sone et al., 2019). Collectively, the convergence of this classic radiological sign, specific histopathological evidence, and definitive genetic confirmation provides a robust foundation for the diagnosis, fully aligning with established criteria and highlighting the importance of considering NIID in patients with atypical neurological syndromes.

Based on the available evidence, this case is best classified as apparently sporadic. This classification is based on: (1) a comprehensive family history negative for relevant disorders, and (2) the absence of the pathogenic expansion in her two tested first-degree relatives (sons). While the autosomal dominant inheritance of NIID is well-established, the possibility of a *de novo* mutation in the proband is a recognized mechanism for apparently sporadic cases in repeat expansion disorders. Although a *de novo* event cannot be formally proven in this instance without testing the parents, this mechanism has been proposed for NIID and is consistent with our findings (Ishiura et al., 2019). Alternatively, incomplete penetrance in a parent, though less documented in NIID, remains a theoretical possibility. Given these considerations, “apparently sporadic” most accurately reflects the current state of knowledge for this pedigree. The patient’s relatively preserved cognitive function, despite a 7-year history of symptoms and clear radiological progression, strongly supports the hypothesis that non-specific symptoms like dizziness and headache can represent a prolonged paucisymptomatic or prodromal stage of NIID. This aligns with longitudinal observations in large NIID cohorts. For instance, Tai et al. described an “episodic neurogenic event-dominant type” of NIID, where patients initially present with paroxysmal events, including headache, often years before the development of overt dementia or motor decline (Tai et al., 2023). The subtle deficits in the visuospatial/executive domain on this patient’s MoCA testing, despite a normal overall score, may represent the nascent sign of evolving cognitive involvement, further underscoring that she is likely in an early phase of the disease.

Previous studies have shown that the characteristic of pathogenic GGC expansion is that the repeat number ranges from 60 to 517. A review has summarized how different repeat sizes may lead to different clinical phenotypes (Zhang et al., 2024): individuals presenting with a myasthenic phenotype typically show larger GGC expansions, often exceeding 200 repeats, with the largest observed expansion reaching 517. These patients also exhibit a higher frequency of GGA trinucleotide interruptions and fewer AGC trinucleotide interruptions. The Parkinson’s disease phenotype, on the other hand, is associated with smaller repeat numbers, usually below 100, with fewer GGA trinucleotide interruptions and more common AGC trinucleotide interruptions. Individuals whose main phenotype is dementia typically show GGC repeat sizes and GGA trinucleotide interruption frequencies within the range of 100–200 repeats. Due to the small number of cases, it is not possible to determine whether there is a connection between GGC expansion numbers and nonspecific symptoms.

## 4 Conclusion

In summary, recurrent dizziness and headache are nonspecific and challenging manifestations of NIID, and the high signal on DWI at the cortical-subcortical junction in the head MRI is an important clue for diagnosis. Clinicians should pay attention to the differential diagnosis with other diseases and are advised to confirm the diagnosis through skin biopsy and genetic testing.

## Data Availability

The datasets presented in this study can be found in online repositories. The names of the repository/repositories and accession number(s) can be found in the article/Supplementary Material.

## Supplementary material

Supplementary Figure S1 Histopathological Findings of NIID. Intranuclear inclusions are observed in Schwann cells and fibroblasts (red arrow). The nuclear inclusions in Schwann cells (**(A)**: 500 nm) and fibroblasts (**(B)**: 500 nm) exhibit unencapsulated fibrous structures.

Supplementary Figure S2 Results of repeat-primed polymerase chain reaction (RP-PCR) analysis of the 5′-untranslated region (5′-UTR) of the *NOTCH2NLC* gene. **(A)** The chromatogram displays a zigzag peak pattern with a gradual decline, indicating the presence of GGC repeat expansion mutations. **(B)** Fluorescence-based PCR amplification reveals 114 GGC repeats at the 5′ end of the *NOTCH2NLC* gene (red arrow).
