# Supplementary figures and images for: Neuronal intranuclear inclusion disease presenting with recurrent dizziness and headache: a case report with 5-year follow-up
Source: Front Genet. 2025 Dec 8;16:1719182. doi: 10.3389/fgene.2025.1719182 (PMC12719417; doi:10.3389/fgene.2025.1719182)

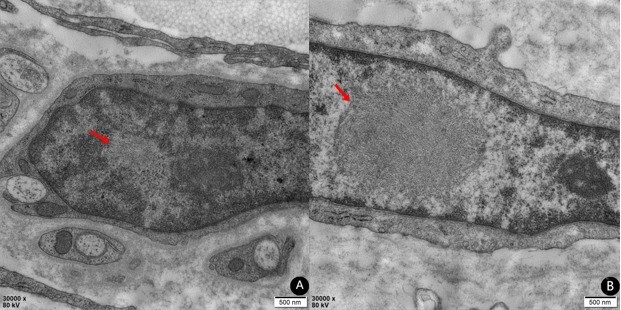

Supplement: Supplementary file 1 [file Image1.jpeg]

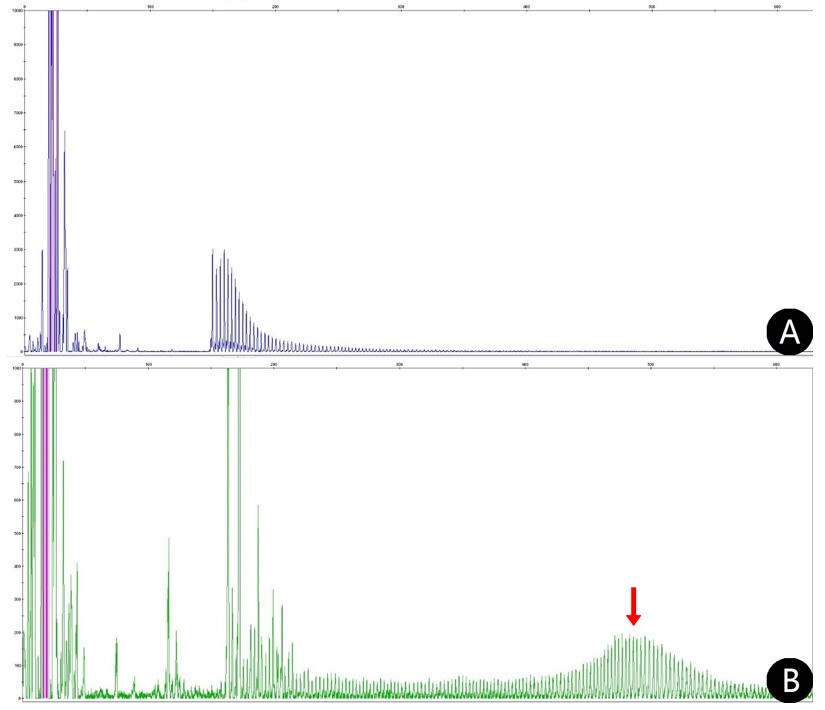

Supplement: Supplementary file 2 [file Image2.jpeg]
